# Metabolic Adaptation as Potential Target in Papillary Renal Cell Carcinomas Based on Their In Situ Metabolic Characteristics

**DOI:** 10.3390/ijms231810587

**Published:** 2022-09-13

**Authors:** Ildikó Krencz, Enikő Vetlényi, Titanilla Dankó, Gábor Petővári, Dorottya Moldvai, Dániel Sztankovics, Regina Raffay, Katalin Mészáros, Endre Sebestyén, Gyula Végső, Judit Pápay, Anna Sebestyén

**Affiliations:** 1 Department of Pathology and Experimental Cancer Research, Semmelweis University, Üllői út 26, H-1085 Budapest, Hungary; 2 HAS-SE Momentum Hereditary Endocrine Tumour Syndromes Research Group, Hungarian Academy of Sciences and Semmelweis University, Üllői út 26, H-1085 Budapest, Hungary; 3 Department of Surgery, Transplantation and Gastroenterology, Semmelweis University, Üllői út 78, H-1082 Budapest, Hungary

**Keywords:** papillary renal cell carcinoma, mTOR, metabolism, in situ expression and in vitro studies

## Abstract

Metabolic characteristics of kidney cancers have mainly been obtained from the most frequent clear cell renal cell carcinoma (CCRCC) studies. Moreover, the bioenergetic perturbances that affect metabolic adaptation possibilities of papillary renal cell carcinoma (PRCC) have not yet been detailed. Therefore, our study aimed to analyze the in situ metabolic features of PRCC vs. CCRCC tissues and compared the metabolic characteristics of PRCC, CCRCC, and normal tubular epithelial cell lines. The protein and mRNA expressions of the molecular elements in mammalian target of rapamycin (mTOR) and additional metabolic pathways were analyzed in human PRCC cases compared to CCRCC. The metabolic protein expression pattern, metabolite content, mTOR, and metabolic inhibitor sensitivity of renal carcinoma cell lines were also studied and compared with tubular epithelial cells, as “normal” control. We observed higher protein expressions of the “alternative bioenergetic pathway” elements, in correlation with the possible higher glutamine and acetate consumption in PRCC cells instead of higher glycolytic and mTOR activity in CCRCCs. Increased expression of certain metabolic pathway markers correlates with the detected differences in metabolite ratios, as well. The lower lactate/pyruvate, lactate/malate, and higher pyruvate/citrate intracellular metabolite ratios in PRCC compared to CCRCC cell lines suggest that ACHN (PRCC) have lower Warburg glycolytic capacity, less pronounced pyruvate to lactate producing activity and shifted OXPHOS phenotype. However, both studied renal carcinoma cell lines showed higher mTOR activity than tubular epithelial cells cultured in vitro, the metabolite ratio, the enzyme expression profiles, and the higher mitochondrial content also suggest increased importance of mitochondrial functions, including mitochondrial OXPHOS in PRCCs. Additionally, PRCC cells showed significant mTOR inhibitor sensitivity and the used metabolic inhibitors increased the effect of rapamycin in combined treatments. Our study revealed in situ metabolic differences in mTOR and metabolic protein expression patterns of human PRCC and CCRCC tissues as well as in cell lines. These underline the importance in the development of specific new treatment strategies, new mTOR inhibitors, and other anti-metabolic drug combinations in PRCC therapy.

## 1. Introduction

Kidney cancer accounts for 3–5% of adult malignancies with several distinct subtypes that have different histological appearance, genetic background, and clinical behavior. The most common histological subtypes are clear cell renal cell carcinomas (CCRCCs, representing about 80% of the cases) and papillary renal cell carcinomas (PRCCs, representing about 10–15% of the cases) [1,2]. Most of the metabolic gene and protein expression studies have presented subgroup independent mappings and/or detailed data about CCRCCs. Although PRCC is the second most common kidney cancer type, there is only a little information available about its metabolic features; and these former studies described only some elements in correlation with only single pathway (e.g., glucose uptake), transcriptomic, or other data analyses. The characterization of metabolic protein expression differences in situ highlights the different metabolic protein expression profiles of PRCCs compared to CCRCCs that has not been performed/published yet.

Mutations in kidney cancers frequently affect genes involved in energy- and oxygen-sensing pathways or different metabolic processes (e.g., *VHL*, *MET*, *TSC1/2*, *SDH*, and *FH*). Therefore, regarding the resulting bioenergetic dysregulation of cancer cells [3], kidney cancers are considered neoplasms characterized by metabolic reprogramming [4]. Some of the above-mentioned alterations can affect not only CCRCCs but also PRCCs [5]. These alterations may assist malignant transformation and allow to fulfil the energetic and biosynthetic demands associated with increased proliferation and cell growth [6]. For example, the inactivation of fumarate hydratase (FH) or succinate dehydrogenase (SDH) in some forms of renal cell carcinomas (RCCs) result in the direct interruption of the tricarboxylic acid (TCA) cycle and oxidative phosphorylation (OXPHOS). These alterations oblige the use of aerobic glycolysis (or “Warburg effect”), truncated TCA cycle, and other bioenergetic processes to produce adenosine triphosphate (ATP) and biosynthetic macromolecules in cancer cells [5]. Glucose utilization can support the pentose phosphate pathway (PPP) generating ribose 5-phosphate and NADPH for nucleotide and fatty acid synthesis [7]. In addition, glutamine can be an additional energy or nutrient substrate for protein and lipid synthesis in tumor cells. Glutamine consumption can promote glutathione (GSH) synthesis, the main reactive oxygen species (ROS) scavenger, which was highlighted in a recently published proteome profile analysis of PRCC cases [8]. Glutaminases (GLS) catalyze glutamine deamination, then glutamate can also be converted to α-ketoglutarate which enters the TCA cycle. Glutamine-glutamate via α-ketoglutarate conversion can be used for ATP production and fueling TCA cycle to serve biosynthetic processes [9]. Moreover, other amino acids can also contribute to biomass production and energy derivation in versatile ways via anaplerotic reactions or can be required for glutathione synthesis (Ser, Leu, Asp, Cys, etc.) [10]. Additionally, acetyl-CoA production can also be supplied by the acetyl-CoA synthetase utilizing exogenous acetate [11] or can be produced by β-oxidation of fatty acids [12].

In response to intra- and extracellular signals and nutrient availability, the phosphatidylinositol 3-kinase (PI3K)/protein kinase B (Akt)/mammalian target of rapamycin (mTOR) pathway plays an important role in the regulation of bioenergetic processes, and additionally, it is also frequently hyperactivated in RCCs [13,14]. The serine-threonine kinase mTOR forms the catalytic subunit of two multiprotein complexes, namely mTOR complex 1 (mTORC1) and mTOR complex 2 (mTORC2) [15]. Through its downstream targets (e.g., S6 kinase, eukaryotic translation initiation factor 4E-binding protein, and sterol regulatory element-binding proteins), mTORC1 influences several metabolic processes, including mitochondrial biogenesis, nucleotide, and lipid synthesis [16,17]. The importance of mTORC2 in metabolic reprogramming is less well-characterized, but its role in fatty acid metabolism and glucose homeostasis has already been described [18].

The mTORC1 inhibitors temsirolimus and everolimus have been approved for the treatment of patients with advanced kidney cancer; however, these have not been shown to result in any long-lasting antitumor effect. Readdressing the role of mTOR inhibitors, administration of next-generation agents targeting the mTOR pathway, identification of predictive biomarkers for patient selection, and combination therapies may increase the efficacy of mTOR inhibitor therapy in the treatment of RCCs [19].

Given the mTOR pathway is of paramount importance in regulating the metabolic phenotype, it is conceivable that the efficacy of mTOR inhibitors is at least partly a result of bioenergetic dysregulation [18]. Moreover, identifying the role of metabolic processes in the pathobiology of RCCs has aroused interest targeting bioenergetic pathways which are thought to be crucial for the proliferation and survival of tumor cells. It has been indicated that enzymes involved in glutaminolysis may serve as ideal targets for personalized therapy. Based on this observation, GLS inhibitors have been tested in clinical studies and have shown promising results in metastatic RCCs [20]. The addition of telaglenastat (a GLS inhibitor, also known as CB-839) to everolimus also increased the progression-free survival of heavily pre-treated, metastatic RCC patients in the ENTRATA phase II study in CCRCCs (NCT03163667). These well-known data and experimental results were described in CCRCCs, but the metabolic alterations (glutamine or other alternative nutrient utilization) in non-CCRCCs are less studied. While genetic alterations have been clearly described for hereditary papillary cancers, the metabolic landscape of other PRCCs have not yet been characterized. It was also demonstrated that the lack of functional enzymes in the TCA cycle (e.g., FH or SDH) could increase the glycolytic activity and reliance on both glucose and glutamine for cell proliferation and survival in vitro. However, few data are available about the metabolic features of other mutations or signaling alterations related to PRCCs. For example, a c-MYC-driven mouse model of an aggressive RCC was characterized by increased HIF1α stabilization and glutamine use [21]. Thus, glutamine utilization or other alternative metabolic pathways could be potential drug targets even for PRCCs.

Although advantages of MET tyrosine kinase inhibitors have been described in the treatment of PRCCs recently, conventional therapies seem to be less effective for PRCCs. This is due to the different genetic and biological background as well as the limited number of patients participating in the clinical trials compared to CCRCC patients [22]. Accordingly, the importance of metabolic alterations has been highlighted in the pathobiology of RCCs, as there could be significant differences in the nature of the bioenergetic perturbances and the genetic background among the various forms of RCCs. Elucidating the metabolic phenotypes of histological variants is crucial for the development of specific treatment strategies against a given subtype. In our study, several markers of mTOR activity and certain metabolic pathways were analyzed in non-FH- or -SDH-mutant PRCCs compared with CCRCCs. Additionally, some metabolic and mTOR inhibitors as well as their combinations were also tested in in vitro models.

## 2. Results

### 2.1. Immunohistochemical Expression and Correlations of mTOR Pathway and Metabolism-Related Proteins in Papillary and Clear Cell Renal Cell Carcinomas

**Figure 1:**
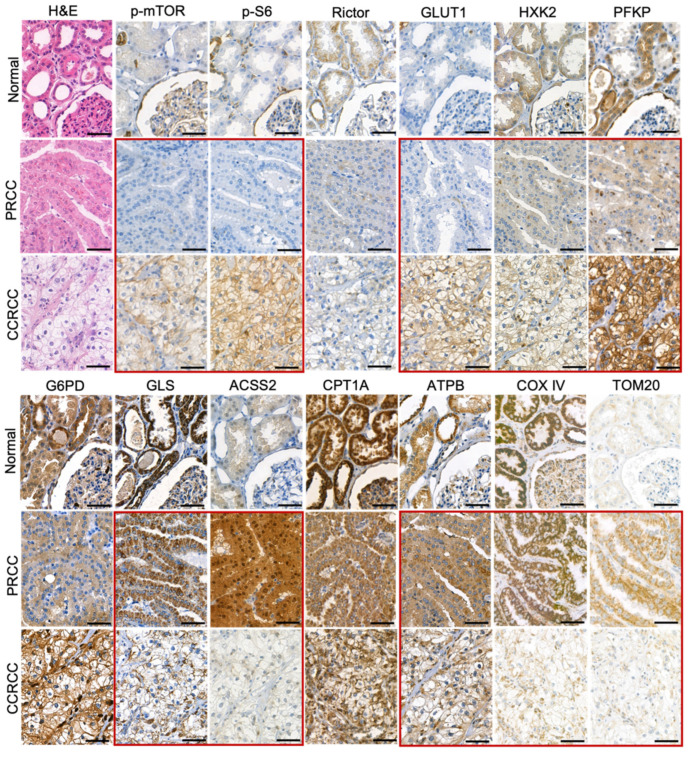
Immunohistochemical analysis of mTOR and metabolic pathway markers in papillary (PRCC) and clear cell renal cell carcinomas (CCRCC). The expression of p-mTOR (marker for mTOR kinase activity), p-S6 (mTORC1 activity marker), Rictor (marker of the amount of mTORC2), GLUT1, HXK2, PFKP (glycolysis markers), G6PD (pentose phosphate pathway marker), GLS (glutaminolysis marker), ACSS2 (marker for acetate consumption), CPT1A (fatty acid β-oxidation marker), ATPB (OXPHOS marker), COX IV, and TOM20 (mitochondrial markers) was analyzed in normal kidney, PRCCs, and CCRCCs. The detected characteristic differences in enzyme expression profiles between PRCCs and CCRCCs were highlighted by red frame. Immunohistochemistry (DAB chromogen–brown) and hematoxylin counterstaining were used. Scale bars indicate 50 μm.

In the normal proximal tubular epithelial cells, we observed low expression of p-mTOR, p-S6, glucose transporter 1 (GLUT1), hexokinase 2 (HXK2), ACSS2, and TOM20. In contrast, the tubular epithelial cells showed moderate or high expression of Rictor, phosphofructokinase (PFKP), glucose-6-phosphate dehydrogenase (G6PD), glutaminase (GLS), carnitine palmitoyltransferase 1A (CPT1A), β-F1-ATPase (ATPB), and COX IV (Figure 1).

PRCCs had no increased in situ GLUT1 expression and in correlation with this neither mTORC1 activity (p-mTOR, p-S6), nor glycolytic HXK2 and PFKP enzymes showed high expression. The most significant difference between PRCC and normal tubular epithelial cells was observed in the expression of ACSS2. The highly increased expression of ACSS2 in PRCCs suggests high tumor-specific acetate consumptions in these cells. Meanwhile, the expected distinction between CCRCC and normal tubular epithelial cells in correlation with the known highly glycolytic properties of CCRCCs were detected in p-mTOR, p-S6, and GLUT1.

**Figure 2:**
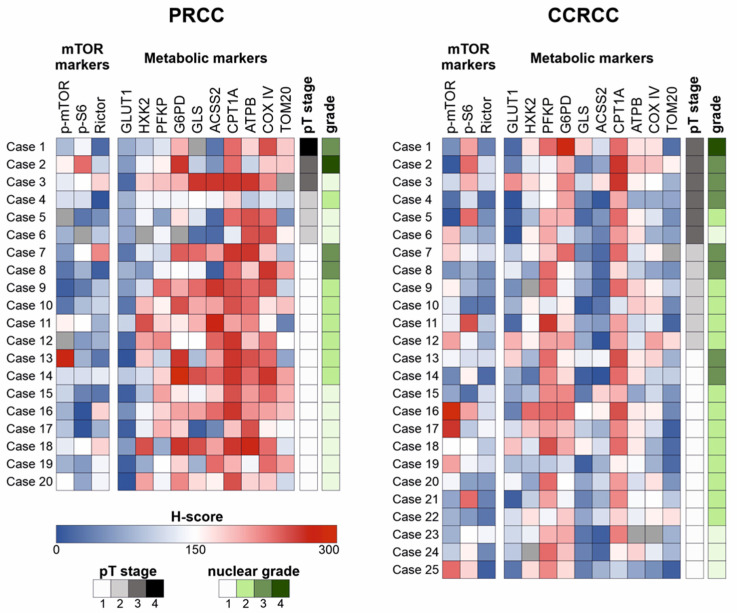
Heat map visualization of the scored in situ expression analyses of mTOR and metabolic pathway markers in papillary (PRCC) and clear cell renal cell carcinomas (CCRCC). Immunohistochemical expression of the markers was visualized on a heat map in each case. Blue color indicates low expression, whereas red color indicates high expression. Missing values are shown in grey. The pT stage and nuclear grade are also available.

The expressions of p-S6 (marker for mTORC1 activation), GLUT1, and PFKP (glycolysis markers) were significantly lower in PRCCs as compared to CCRCCs (*p* < 0.05, *p* < 0.01, and *p* < 0.05, respectively), whereas the expressions of HXK2 and G6PD did not significantly differ between the two subgroups (Figure 1 and Figure 2). In correlation with these, the expression of the studied enzymes in alternative bioenergetic pathways, such as GLS (glutaminolysis), ACSS2 (acetate utilization), and CPT1A (fatty acid β-oxidation) was significantly higher in PRCCs as compared to CCRCCs (*p* < 0.01, *p* < 0.01 and *p* < 0.01, respectively). In addition, the higher ATPB expression in PRCCs (*p* < 0.01) with the increased staining intensity of COX IV and TOM20 antibodies also suggest the higher mitochondrial oxidative capacity in papillary subtype.

**Figure 3:**
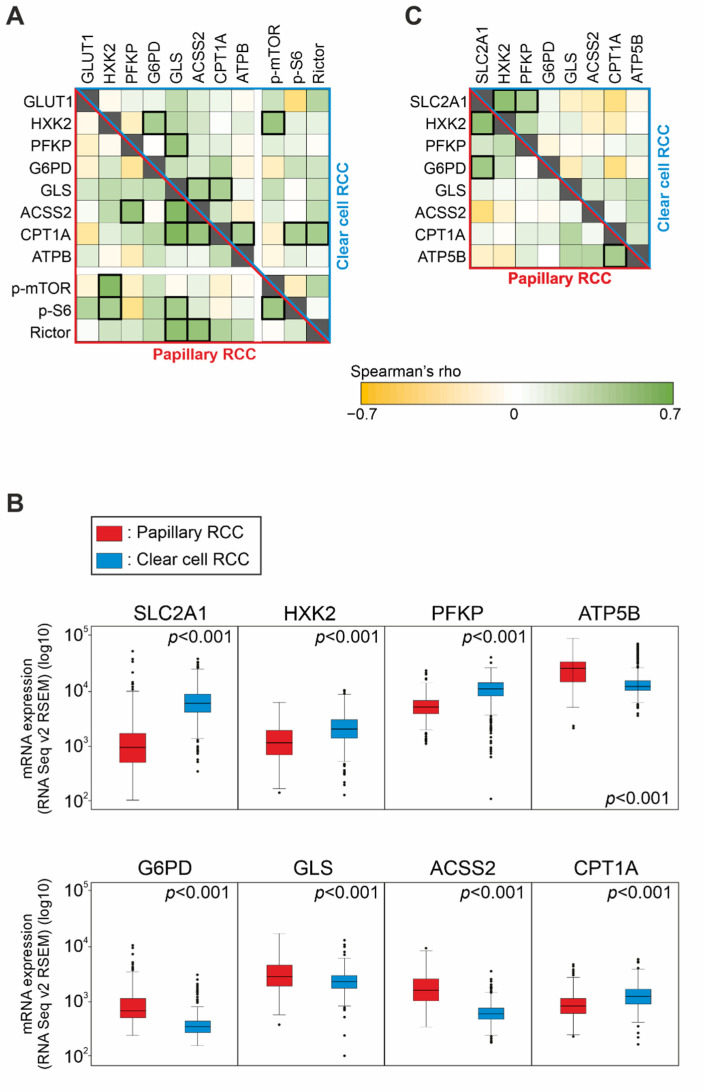
Expression and correlation of the mTOR and metabolic pathway markers in papillary (PRCC) and clear cell renal cell carcinomas (CCRCC). (**A**) Correlation between the protein expression of GLUT1, HXK2, PFKP (glycolysis markers), G6PD (pentose phosphate pathway marker), GLS (glutaminolysis marker), ACSS2 (marker for acetate consumption), CPT1A (fatty acid β-oxidation marker), ATPB (OXPHOS marker), p-mTOR, p-S6, and Rictor (mTOR markers) in papillary (lower, red triangle) and in clear cell (upper, blue triangle) RCCs based on our IHC analysis. Green color indicates a negative, yellow color labels a positive correlation. Strong correlations (Spearman’s R > 0.4 and *p* < 0.05) are marked with a thick outline. (**B**) The mRNA expression of metabolic pathway markers in papillary and clear cell RCCs according to the TCGA KIRP (PRCC) and KIRC (CCRCC) datasets. SLC2A1 and ATP5B are mRNAs coding for GLUT1 and ATPB, respectively. Two-sided *p* values were considered statistically significant below 0.05 (Mann–Whitney U test). (**C**) Correlation between the mRNA expression of metabolic pathway markers in papillary (lower, red triangle) and clear cell (upper, blue triangle) RCCs according to the TCGA datasets. Green color indicates a negative, yellow color labels a positive correlation. Strong correlations (Spearman’s R > 0.4 and *p* < 0.05) are marked with a thick outline.

The H-scores of examined markers were compared with performing statistical analysis in the two carcinoma subtypes separately. A strong positive correlation (Spearman’s R > 0.4 and *p* < 0.05) was observed between p-mTOR and HXK2 expressions in both PRCC and CCRCC cohorts. Additionally, PRCCs showed positive correlations between Rictor–GLS and Rictor–ACSS2, as well as between p-S6–GLS in the studied cases. Moreover, an association was found between the alternative metabolic pathway markers (GLS, ACSS2, and CPT1A) in both cohorts (Figure 3A).

Regarding the clinicopathological parameters, there were no prognostically relevant findings in PRCCs; however, the expression of G6PD was higher in younger patients (*p* < 0.05). In CCRCCs, G6PD expression was significantly higher in males as compared to females (*p* < 0.05), and higher expression of p-mTOR and CPT1A was associated with lower nuclear grade (*p* < 0.05).

### 2.2. mRNA Expression of Metabolic Pathway Markers in Papillary and Clear Cell Renal Cell Carcinomas

We analyzed the data from the publicly available datasets TCGA KIRP (PRCC cases) and TCGA KIRC (CCRCC cases) and found a similar tendency regarding the mRNA expression of the same metabolic markers that we observed by IHC.

According to the above-mentioned IHC results, mRNA expression of G6PD, GLS, ACSS2, and ATP5B (coding for ATPB) were significantly higher in PRCCs (*p* < 0.01) compared to CCRCCs. Additionally, SLC2A1 (coding for the protein GLUT1), HXK2, and PFKP were significantly lower in PRCCs (*p* < 0.01) that suggest a lower glycolytic capacity in this subtype. In contrast to the IHC results, mRNA expression of CPT1A showed lower level in PRCCs than in CCRCCs (*p* < 0.01) (Figure 3B).

Analyzing the associations among mRNA expressions, a strong positive correlation (Spearman’s R > 0.4 and *p* < 0.01) was observed between SLC2A1 and G6PD expressions as well as CPT1A and ATP5B expressions in PRCCs. This is in correlation with our in situ protein expression results suggesting lower glycolytic and higher mitochondrial OXPHOS activity in PRCCs. We also found a strong positive correlation between the glycolytic markers SLC2A1 and HXK2 in both datasets. Furthermore, SLC2A1 and PFKP also correlated in CCRCCs (Figure 3C). This is in line with previously published highly glycolytic metabolic features of CCRCCs.

In an additional analysis, we found higher mRNA expression of GLS and CPT1A as well as SLC2A1, GLS, and G6PD in women in the PRCC and CCRCC cohorts, respectively (*p* < 0.05). Moreover, higher mRNA expression of G6PD was associated with higher pT stage (*p* < 0.01) in both datasets, and higher expression of SLC2A1 was also observed in cases with higher pT stage (*p* < 0.01) in the CCRCC cohort. According to our IHC results, higher mRNA expression of CPT1A was associated with lower nuclear grade, as well (*p* < 0.05).

### 2.3. Expression of mTOR and Metabolic Pathway Markers in Papillary, Clear Cell Renal Cell Carcinoma, and Tubular Epithelial Cell Lines

**Figure 4:**
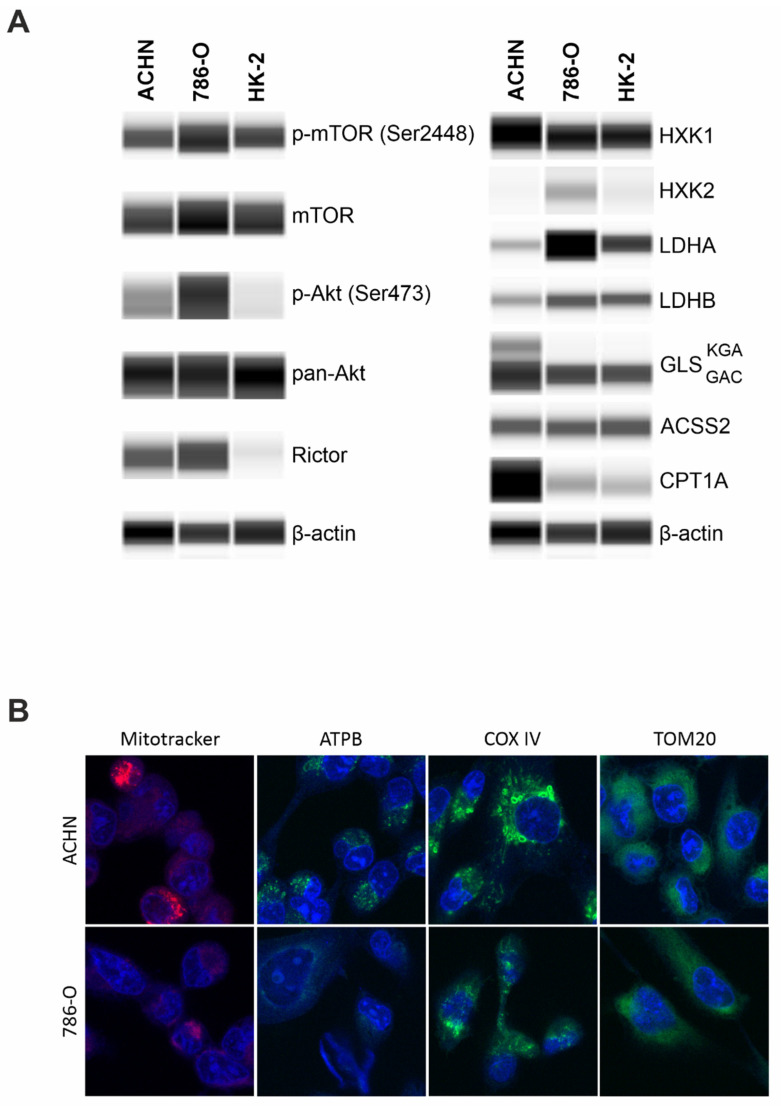
WES analysis of mTOR activity, metabolic enzyme expressions, and in situ mitochondrial stainings of papillary (ACHN) and clear cell (786-O) renal cell carcinoma cell lines. (**A**) WES analysis of mTOR pathway and metabolic markers in papillary (ACHN), clear cell renal cell carcinoma (786-O), and normal tubular epithelial (HK-2) cell lines. β-actin was used as loading control. GLS levels were analyzed by a dual-specificity antibody showing both the KGA (60 kDa) and GAC (55 kDa) splice variants. Predicted protein sizes are provided in Table 2. (**B**) Higher staining intensity with Mitotracker, anti-ATPB, anti-COX IV in ACHN (PRCC) than 786-O (CCRCC) cell lines suggest pronounced mitochondrial OXPHOS function in PRCC cells; however, TOM20 stainings showed no differences (magnification 63×).

Papillary (ACHN), clear cell renal cell carcinoma (786-O), and the immortalized human proximal tubular epithelial cell line (HK-2) exhibited similar p-mTOR levels that indicate a significant mTOR kinase activity in all the studied cells. Besides mTORC1 activity, higher levels of p-Akt (downstream target of mTORC2) and Rictor (scaffold protein of mTORC2) propose a characteristic mTORC2 activity in carcinoma cell lines (ACHN and 786-O), as well. In the non-malignant immortalized tubular epithelial HK-2 cells, we observed a significant p-mTOR level coupled with low or practically no p-(Ser473)-Akt and Rictor expressions. These data show balanced mTORC1 and C2 activity in carcinoma cell lines, and mTORC1 preference in HK-2 cells (Figure 4A).

Besides a slight elevation of hexokinase 1 (HXK1) level, higher levels of alternative metabolic pathway markers, such as GLS and CPT1A and lower expression of Warburg glycolytic markers were observed; both LDHA/LDHB ratio and HXK2 expression were lower in ACHN (PRCC) compared to 786-O (CCRC). Additionally, this difference appeared also if we cultured 786-O at similar conditions to ACHN and HK2 cells, the higher glucose concentration (in high glucose DMEM) did not significantly alter the metabolic enzyme expression profiles of 786-O cells (supplementary data Figure S1). These observations suggest significant differences in the metabolic phenotype among the analyzed cell lines. Moreover, ACHN expressed both isoforms of GLS (kidney-type glutaminase (KGA) and glutaminase C (GAC)), while 786-O and HK-2 expressed only the GAC isoform (Figure 4A). The increased CPT1A and both isoforms of GLS KGA/GAC suggest altered nutrient utilization with lowered Warburg glycolytic phenotypes and OXPHOS metabolic shift in PRCCs vs. CCRCCs. To underline these differences detected by WES, significantly higher ATPB, COX IV, and Mitotracker fluorescent immunostainings confirmed a potentially higher OXPHOS intensity and higher number of mitochondria in PRCC cells compared to CCRCC cell line (Figure 4B).

The detected differences in the cell lines underline the in situ described expression differences and their potential role in papillary vs. clear cell type-dependent metabolic adaptation mechanisms.

### 2.4. Intra- and Extracellular Metabolite Concentrations in Papillary, Clear Cell Renal Cell Carcinoma, and Tubular Epithelial Cell Lines

The measured intra- and extracellular metabolite concentrations (the lactate/pyruvate, lactate/malate, pyruvate/citrate, and lactate/glutamate ratios) helped to characterize the differences in the activity of metabolic processes in the HK-2 and both types of carcinoma cell lines.

**Figure 5:**
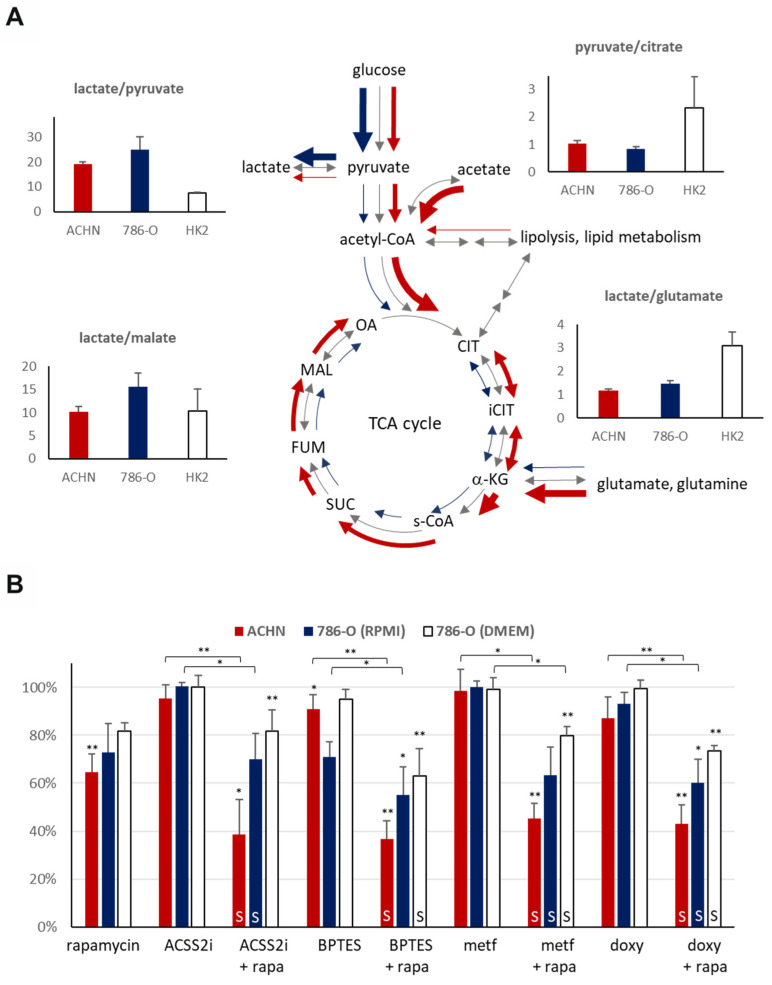
LC–MS analyses of metabolic features and inhibitor sensitivity of papillary (ACHN) and clear cell renal cell carcinoma (786-O) cell lines. (**A**) LC–MS analysis of the intracellular metabolite concentrations in papillary (ACHN), clear cell (786-O) RCC and normal tubular epithelial (HK-2) cell lines. Lactate/pyruvate, pyruvate/citrate, and lactate/malate ratios were used to assess the glycolytic capacity and the activity of the TCA cycle of the cell lines (CIT–citrate, iCIT–isocitrate, α-KG–alpha-ketoglutarate, s-CoA–succinyl-CoA, SUC–succinate, FUM–fumarate, MAL–malate, OA–oxaloacetate), whereas lactate/glutamate ratio was used to evaluate the glutamine utilization. (**B**) The mTORC1 (rapamycin–rapa) and metabolic inhibitors (ACSS2i–inhibitor of acetate utilization, BPTES–glutaminolysis inhibitor, metformin–metf–AMPK-inhibitor, doxycycline–doxy–antibiotics with mitochondrial inhibitory effect) sensitivity of papillary (ACHN) and clear cell (786-O) RCC cell lines were evaluated as monotherapy or in combination. Regarding the 786-O cells, the treatments were performed both in RPMI-1640 (2000 mg/L glucose) medium under generally applied and optimized conditions and in DMEM high glucose (4500 mg/L)–to compare the role of maintaining parameters with similar glucose concentration levels as ACHN and HK-2 cells. * *p* values below 0.05, ** *p* values below 0.01 (one-way ANOVA with Tukey’s post hoc test), and at least 20% decrease in proliferation were considered biologically relevant. Synergistic treatment interactions were labeled with S (based on combination index calculation).

The intracellular lactate/pyruvate ratio was approximately two to three-fold higher in the RCC cell lines (786-O: 24.80 [SD: 5.32], ACHN: 19.26 [SD: 0.68]) as compared to HK-2 tubular epithelial cells (7.56 [SD: 0.29]), which shows that the higher glycolytic capacity is associated with higher in vitro proliferation rates of the carcinoma cells (Figure 5A). In correlation with the detected differences between ACHN and 786-O in lactate/pyruvate ratio underline the lower Warburg glycolytic capacity of PRCCs in ACHN at previously optimized cell culture conditions using DMEM with high glucose (4500 mg/L). This can also be confirmed by the lower pyruvate/citrate and higher lactate/malate ration (intra- and extracellular) in 786-O (CCRCC cancer cells) even in RPMI-1640 media with lower glucose concentration (2000 mg/L). The extracellular lactate/pyruvate ratio difference in the RCC cell lines (ACHN: 11.71 [SD: 0.28], 786-O: 7.32 [SD: 0.50]) could be explained by the relative decrease in pyruvate concentration of ACHN. This correlates with increased pyruvate shift to TCA and enhances OXPHOS (without higher lactate production) in the ACHN cell line. Moreover, as compared to the high-glucose dependent HK-2 cells (growing in DMEM high glucose), we observed an almost three-fold lower intracellular lactate/glutamate ratio in the studied carcinoma cells, and approximately comparable amount of intracellular lactate to glutamate in cancer cells, especially in PRCCs (ACHN: 1.16 [SD: 0.07], 786-O: 1.45 [SD: 0.13] vs. HK-2: 3.09 [SD: 0.57]). It raises the possibility that these cells can uptake/fuel and consume extracellular glutamine and use glutamate (as energy source), ROS scavenger, and biosynthetic building blocks at high level. In line with this observation, we found smaller differences in extracellular lactate/glutamate ratio between 786-O and HK-2 cells (6.99 [SD: 2.11] and 7.49 [SD: 0.50], respectively); however, ACHN cells showed significantly higher extracellular lactate/glutamate ratio (39.54 [SD: 3.37]) that suggest a relatively higher glutamate consumption in PRCC cells in vitro.

### 2.5. The Effect of mTOR and Metabolic Inhibitors on the Proliferation of Papillary and Clear Cell Renal Cell Carcinoma Cell Lines

SRB assay was performed to analyze the antiproliferative effect of the mTORC1 inhibitor (rapamycin), the ACSS2 inhibitor (ACSS2i), the GLS inhibitor (BPTES), the OXPHOS inhibitor (metformin), and the mitochondrial function inhibitory antibiotic (doxycycline) on the studied cell lines. Different cell culturing conditions (such as glucose concentration) could alter the viability and proliferation capacity of renal cells [23]. Therefore, we tested the studied treatments using the DMEM high glucose media (similar culturing conditions to HK-2 and ACHN) in case of 786-O CCRCC cells. The different glucose concentration altered the GLS inhibitor sensitivity (the cells were less sensitive to monotherapies); however, the rapamycin + BPTES and doxycycline + rapamycin combinations have almost similar effects. The effects of treatments were also tested in EMEM (1000 mg/L glucose) in case of the ACHN PRCC cell line. The effects of the studied treatments and their combinations were almost similar in DMEM high glucose and EMEM-cultured ACHN cells (data not shown), in harmony with the findings of Morais C. et al., showing that the effects of various treatments could not be influenced by applying a high glucose concentration in renal proximal tubular cells [24].

Rapamycin had a significant antiproliferative effect (decreased cell amount with 30–40%) in the studied carcinoma cell lines and only a slight effect on the HK-2 cells in vitro at applied different conditions. It was surprising that the ACHN (PRCC) cell line was resistant to all metabolic inhibitor monotherapies (ACSS2 inhibitor–ACSS2i, BPTES, metformin, and doxycycline). The 786-O (CCRCC) was GLS inhibitor (BPTES) sensitive only (the proliferation decreased to ~71%) while being generally applied and optimized by maintaining conditions (in RPMI-1640 medium). These observations underline the potential importance of glutamine utilization in RCCs and their targeted therapy.

Metabolic inhibitors and their rapamycin combinations slightly influenced the proliferation and survival of HK-2 cells (proliferation rate remained between 80–100%, data not shown). However, the studied carcinoma cell lines were mainly resistant to metabolic inhibitor monotherapies (except for rapamycin); rapamycin combination treatments could increase the sensitivity and significantly inhibit the proliferation. We observed a higher synergism of rapamycin plus metabolic inhibitor combinations in ACHN; these effects were less pronounced in 786-O (not all combinations showed a significant decrease) (Figure 5B). Additionally, the renal toxicity of these combinations needs to be further analyzed in primary isolated or other kidney tubular epithelial cells (such a RPTEC cell line), as well as in animal experiments.

## 3. Discussion

Kidney cancer has already been described as a so-called “metabolic disease”; however, studies have focused primarily on CCRCCs, which represent the majority (~80%) of RCC cases [1,2,3]. Metabolic reprogramming in other subtypes, such as PRCCs, has not been extensively characterized; to our knowledge, our study is the first comparative in situ analysis of the metabolic enzyme expression profile of PRCCs and CCRCCs, including human samples.

**Figure 6:**
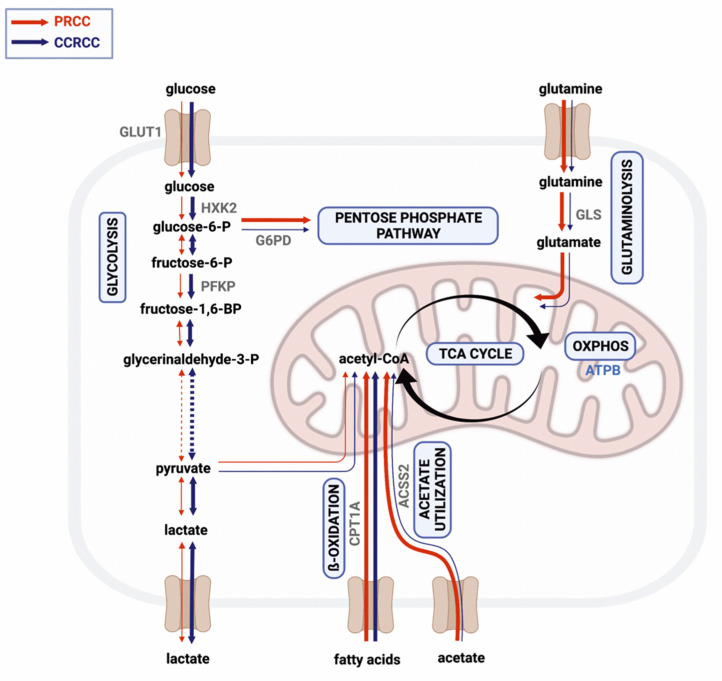
Simplified scheme of the metabolic pathways analyzed in papillary (PRCC) and clear cell renal cell carcinoma (CCRCC). Red and blue arrows on left and right side of black ones indicate the characteristic rewiring of metabolic pathways in PRCCs and in CCRCCs, respectively. High expressions of certain enzymes suggest balanced nutrient utilization, intensive glutaminolysis (GLS), acetate utilization (ACSS2), fatty acid oxidation (CPT1A), and oxidative phosphorylation (ATPB) were detected in PRCCs (thick red arrows), while high expressions of glycolytic enzymes (GLUT1, HXK2, PFKP) could correlate with higher glycolytic capacity in CCRCCs (thick blue arrows). Created with BioRender.com.

Dependency on glycolysis has already been revealed in RCC [25], especially in CCRCC cases harboring *VHL* mutation that has an important role in the regulation of the oxygen-sensing pathways [3]. Consistent with the already published data, we observed higher expression of glucose transporter and glycolytic enzymes in CCRCCs as compared to PRCC cases. However, the detected GLUT1 expression in PRCCs was not higher than in non-malignant renal tissues and was definitively lower than in CCRCC cases in contrast to the observation described by Almeida about glucose transporter expressions [26]. Additionally, we detected higher expression of enzymes related to alternative metabolic pathways, such as glutaminolysis and acetate utilization in PRCCs (Figure 6). To strengthen our results, we also analyzed the mRNA expressions of the same markers in a larger cohort of RCC cases using a public database. Except for CPT1A, we found similar expression differences between the two subtypes that were observed in our results at protein level. These and the detected high expression of ATPB, GLS, and ACSS2 in the PRCC cases suggest that acetate and glutamine may primarily fuel the TCA cycle influencing both OXPHOS and lipid metabolism in this subtype.

Based on these, our IHC and in vitro cell line characterization data suggest that PRCC cells have survival and progression benefit through their balanced metabolic phenotype with considerable mTORC2, mitochondrial OXPHOS activity, as well as high glutamine and acetate utilization [27]. It was described that the higher expression of G6PD can promote proliferation, maintain NADPH level, and redox homeostasis [28]. The increased expression of this enzyme was described in 10 PRCC cases vs. normal kidney [29], as we also detected similarly higher expression level of G6PDH both in PRCC and CCRCC cases.

Ayham Al Mahad performed proteome profile analyses (with ~8000 proteins) of 19 PRCCs; accordingly, they described reduced metabolic activity and Warburg glycolytic shifts in PRRCs compared to normal kidney [8]. In contrast, our analyses detected high level of ATPB and many other mitochondrial proteins including, e.g., TOM20 and COX IV in PRCC cells in in situ tumor tissues. Additionally, increased Mitotracker, ATPB, and COX IV stainings, elevated GLS, CPT1A expression were also detected in PRCC vs. CCRCC cells in vitro. The differences between our study and the mentioned proteome-metabolome analysis results could be explained by the used tissue extracts vs. tissue slides and cells. The tissue heterogeneity (normal, endothelial and other non-tumor cells, ECM elements), the source of the detected changes could not be determined in proteome profile analyses using tissue mass. Additionally, the metabolic heterogeneity of tumor tissues, the known metabolic symbiosis [18] cannot be followed, the tumor- and other cell-derived alterations cannot be distinguished. Our pathomorphology studies with expert pathologists allow to identify malignant and non-malignant compartments and evaluate their expression in situ. Additionally, the in vitro detected metabolic inhibitor sensitivity studies could also correlate to the detected alterations in human PRCC tissues. Our results, data from metabolite concentration ratio analyses and the expression differences of GLS also confirm the importance of potential glutamine/glutamate utilization, and its ROS scavenging effect. Additionally, it highlights other possibilities, as the use of glutamine in TCA fueling and biosynthetic sources [30].

Our results showed a strong positive correlation between the expression of metabolic and mTOR activity markers. This observation agrees with recent studies showing that mTOR has an important role in the regulation of bioenergetic processes [18,31,32]. Association between mTOR pathway and metabolic processes suggests the potential benefit of combining mTOR and metabolic inhibitors. The synergism of mTOR and different metabolic inhibitors has been described in several tumor types [33,34], including CCRCCs, in which the combination of everolimus and GLS inhibitor (CB-839) was also tested [20]. The efficacy of these combinations may rely on the metabolic plasticity of the tumor cells, which can be targeted to inhibit metabolic adaptation and drug resistance [18,33,35]. Correspondingly, the expression of alternative metabolic pathway markers in the studied RCC cases highlights the importance of identifying rational drug combinations which target more (or all) of the activated bioenergetic pathways to achieve metabolic catastrophe with synthetic lethality in cancer cells [35,36]. These combinations could have special importance in advanced PRCCs where the potential mTORC2 activity and the use of glutamine, lipids and/or other nutrient sources could be a basis of metabolic adaptation in cancer progression.

In our in vitro analysis, we found higher mTOR (especially mTORC2) activity and metabolic marker expressions in carcinoma cell lines as compared to the HK-2 cells, according to the higher proliferation capacity of the cancer cells [37]. Moreover, higher lactate/pyruvate and lower lactate/glutamate intracellular metabolite ratios indicated higher glycolytic capacity and glutamine uptake in the RCC cell lines. Based on our LC–MS and WES analyses, high glycolytic activity was detected in the *VHL*-mutant 786-O CCRCC cell line, as it has also been described previously [38]. In contrast, the metabolic profiles of ACHN cells (isolated from metastatic PRCC) were similar to in vivo growing PRCCs detected in patient biopsies.

Interestingly, both variants of the GLS enzyme (KGA and GAC) were detected in ACHN cells by a dual-specificity antibody, while 786-O and HK-2 cell lines expressed only the GAC isoform. KGA is considered the full-length GLS, whereas GAC is a C-terminal truncated splice variant [39]. Both the KGA and GAC isoforms are implicated in cancer; however, the GAC/KGA ratio has been described as higher in most tumor types. Although a correlation between GAC expression and CB-839 sensitivity has been reported in breast cancer [40], our results are consistent with a recent publication reporting that antibodies which cannot distinguish GLS isoforms at tissue level (by IHC) are not suitable for studying potential GLS sensitivity in patients’ biopsies [41]. The majority of carcinoma cell lines are resistant to BPTES as monotherapy. 786-O cells (expressing only the GAC isoform) showed a slightly higher antiproliferative effect (at generally applied optimized conditions) of BPTES monotherapy while ACHN was practically resistant to this treatment. However, synergism of rapamycin and BPTES combination treatment was detected in both RCC cell lines in our in vitro treatments. These results propose that splice variants of GLS may have a role in altered sensitivity to the most promising GLS inhibitors in RCC treatment [20], based on our results in PRCC cells. Moreover, the detected GLS isoforms may not significantly influence the efficacy of mTOR and GLS inhibitor combination, which has significant antiproliferative potential in RCC cell lines. 

ACSS2 generates acetyl-CoA both in the cytosol and in the nucleus for bioenergetic processes and histone acetylation, thereby influencing the metabolism and gene expression of the tumor cells [42]. Targeting ACSS2, that is linked to aggressive clinical behavior and poor prognosis in RCC, may also represent a new therapeutic option [43,44]. Although inhibition of ACSS2 has been shown to result in decreased growth of breast cancer [45], it was shown to significantly decrease the proliferation of the RCC cells only in rapamycin combinations in our in vitro study.

In conclusion, our study underlines the metabolic differences between PRCCs and CCRCCs, and highlights the balanced metabolic activity of these tumors, especially in PRCCs. Based on our analyses, the human PRCCs are less glycolytic and mainly have the OXPHOS phenotype, glutamine, and other TCA-fueled nutrients dependent on metabolism. This metabolic configuration in cancer tissue could help in metabolic adaptation during disease progression. Our results as well as the well-known mTOR activity and the potential mTORC2 activity in PRCCs call the attention to the relevance of combining mTOR and other metabolic inhibitors since BPTES, ACSS2 inhibitors, metformin, and doxycycline were able to enhance the antiproliferative effect of rapamycin depending on the metabolic enzyme expression differences. These presented new results reveal metabolic differences between the most common histological subtypes of kidney cancer and highlight the importance of their metabolic characteristics, which may help to find new druggable targets and develop rational drug combinations for the personalized therapy of PRCCs.

## 4. Materials and Methods

### 4.1. Patients–Tissue Microarray Construction

Metabolic characterization was performed from formalin-fixed, paraffin-embedded tissues of patients diagnosed with PRCC (N = 20) and CCRCC (N = 25). Due to the association between transplantation and RCCs, our study included samples from post-transplant patients (4 PRCC and 2 CCRCC). Immunohistochemistry (IHC) analysis did not detect FH or SDHB protein loss in the selected RCCs. None of the patients received mTOR inhibitor therapy prior to surgery. Therefore, metabolic enzyme mutations and/or treatment-specific alterations were excluded from this study. Samples from donor kidneys, which were considered unacceptable for transplantation due to surgical reasons, were also examined as normal kidney samples (N = 3). Clinicopathological data were collected from medical records, as shown in Table 1. The tumors were reclassified according to the 2016 WHO Classification. The tissue samples were used with the approval of the Scientific Research Council National Ethics Committee for Scientific Research (SE KREB-216/2020). Representative tumor areas for constructing tissue microarrays (TMAs) were selected by pathologists based on hematoxylin and eosin-stained slides. TMA blocks containing duplicate or triplicate cores (of 2 mm diameter) from the same tissue samples were created using TMA Master (3DHistech, Budapest, Hungary). Non-neoplastic kidney and liver samples were used as negative controls.

### 4.2. Immunohistochemistry

After deparaffinization and blocking of endogenous peroxidases, antigen retrieval was applied using citric acid buffer (pH = 6.0) for 30 min. in a pressure cooker. The TMA sections were incubated with primary antibodies shown in Table 2. The reaction was visualized by the Novolink Polymer (Leica Biosystems, Wetzlar, Germany) secondary detection system, and 3,3′-diaminobenzidine (DAB, Dako, Carpinteria, CA, USA) was used as chromogen with hematoxylin counterstaining.

The immunostained slides were evaluated by two independent investigators using CaseViewer software (version: 2.3, 3DHistech). H-score was calculated by a semi-quantitative assessment (scores: 0–300) of the staining intensity (0, 1+, 2+, or 3+) and the percentage of immunopositive cells, as has been described previously [33].

### 4.3. Data Collection from the Cancer Genome Atlas

RNA Seq_v2 and clinicopathological data of PRCCs (N = 277, KIRP–Kidney Renal Papillary Cell Carcinoma cohort) and CCRCCs (N = 502, KIRC–Kidney Renal Clear Cell Carcinoma cohort) were collected from The Cancer Genome Atlas (TCGA, PanCancer Atlas) and downloaded through cBioPortal (www.cbioportal.org, accessed on 15 July 2019) [46]. Cases with FH, SDHx, or TFE3 gene alterations known as putative drivers were excluded from both cohorts. Clinicopathological data, including age, sex, and T stage are summarized in Table 1 (not all parameters were provided in each case).

### 4.4. Cell Cultures, Reagents, and Sulforhodamine B Proliferation Assay

The human PRCC (ACHN), human CCRCC (786-O), and immortalized human kidney proximal tubular epithelial (HK-2) cell lines were cultured and treated in high glucose Dulbecco’s Modified Eagle’s Medium (BioSera, LM-D1108, Nuaille, France) or RPMI-1640 (BioSera, LM-R1641, in case of 786-O) media (Merck–Sigma-Aldrich, Darmstadt, Germany) in the in vitro studies. The media were supplemented with 10% fetal bovine serum (FBS, BioSera), 2 mM L-glutamine (BioSera), and 100 UI/mL penicillin–streptomycin (BioSera), or 40 mg/mL gentamycin (Sandoz, Holzkirchen, Germany).

A total of 2500–3000 cells per well were plated into 96-well plates and after 24 h the media were replaced for different treatments. The mTORC1 inhibitor rapamycin (50 ng/mL, Merck–Sigma-Aldrich), acyl-coenzyme A synthetase short-chain family member 2 (ACSS2) inhibitor (15 µM, Selleckchem Chemicals, Houston, TX, USA), bis-2-(5-phenylacetamido-1,3,4-thiadiazol-2-yl)-ethyl sulfide (BPTES) (5 µM, Merck–Sigma-Aldrich) GLS inhibitor, metformin (300 µM, Merck–Sigma-Aldrich) OXPHOS inhibitor and doxycycline (10 µM, Merck–Sigma-Aldrich) mitochondrial function inhibitory antibiotic were added as monotherapy and in combination with rapamycin for 72 h. Inhibitor concentrations were determined based on our previous studies and other publications [33,47,48,49].

Treatment effectivity was measured by sulforhodamine B (SRB) proliferation assay. Cells were fixed by cold 10% trichloroacetic acid for 60 min at 4 °C and washed. After drying, the cells were incubated with 0.4% SRB (Merck–Sigma-Aldrich) in 1% acetic acid for 15 min at room temperature. The protein-bound dye was dissolved in 10 mM Tris base solution (Merck–Sigma-Aldrich) after 1% acetic acid washing steps. The absorbance at 570 nm was measured with LabSystems Multiskan RC/MS/EX Microplate Reader (Labsystem International; Transmit Software version 4.5—Vantaa, Finland). The relative cell proliferation was calculated as the percentage of untreated cells from three independent experiments using six parallels.

The combination treatments were evaluated by using combination index (*CI*) calculation method with the following formula, where *E_a_* and *E_b_* were the detected effect of individual mono-treatments and *E_ab_* represents the effect of the combination. Depending on the calculated value of *CI,* the effects of combination treatments were determined as synergistic (*CI* < 1), additive (*CI* = 1) or no additional effects (*CI* > 1) [50].

### 4.5. Protein Expression Analysis in Cell Lines

Protein expression analyses were performed using Wes^TM^ Simple capillary Western immunoassay technique (WES). Cells were washed with phosphate-buffered saline and proteins were extracted with lysis buffer (50 mM Tris, 10% glycerol, 150 mM NaCl, 1% Nonidet-P40, 10 mM NaF, 1 mM phenylmethylsulfonyl fluoride, 0.5 mM Na_3_VO_4_, pH = 7.5). Protein concentrations were quantified using the Bradford method (Bio-Rad Laboratories, Hercules, CA, USA).

WES was performed using the Wes^TM^ System (ProteinSimple, San Jose, CA, USA) with a 12–230 kDa Separation Module (ProteinSimple SM-W004), as previously described [34]. Samples were diluted in Sample Buffer (ProteinSimple) at final concentration of 0.2 or 1 µg/µL depending on the applied primary antibody and Fluorescent Master Mix (ProteinSimple) containing dithiothreitol. Samples were denatured, the primary antibodies (Table 2, 1:50 dilution) were applied with the relevant secondary antibodies (Anti-Rabbit Detection Module (ProteinSimple DM-001), Anti-Mouse Detection Module (ProteinSimple DM-002) or Anti-Mouse IgG, HRP-linked Antibody (#7076, Cell Signaling Technologies, Danvers, MA, USA)) and the chemiluminescent substrate (ProteinSimple). The luminol/peroxide chemiluminescence detection was recorded in 1–512 s time frame. The electropherograms were quantified using the instrument’s peak detection with manual correction. The original and unadjusted images of WES analyses can be found in supplementary documentation (Figure S1).

Fluorescent stainings were performed on the studied cell lines using culture slides (Cell Culture Slide, 8 well, SPL Life Sciences) and Mitotracker CMXRos (1:10000, #M7512, Invitrogen, Waltham, MA, USA) or immunolabelling by anti-ATPB (1:100, #ab14730; Abcam), anti-COX IV (1:250, #4850, CST), anti-TOM20 (1:500, #42406, CST) primary antibodies, subsequent fluorescent secondary antibody (A21202-anti-mouse-FITC and A21206-anti-rabbit-FITC, ThermoFisher) and DAPI nuclear stainings. The fluorescent images were analyzed using fluorescent microscopy, (Zeiss LSM780. Jena, Germany—Zen Software version 3.6, Jena, Germany or Nikon Eclipse E600, Nikon Corporation, Tokyo, Japan—Lucia Cytogenetics Software version 3.1, Laboratory Imaging, Prague, Czech Republic, magnification 63×).

### 4.6. Liquid Chromatography–Mass Spectrometry

Intra- and extracellular metabolite (lactate, pyruvate, citrate, malate, and glutamate) analysis was performed as described previously [34]. At least 5  × 10^5^ cells were quenched in liquid nitrogen, then the metabolites were extracted from both cells and supernatants. Chromatographic separations were performed using Luna Omega C18 column (100 × 2.1 mm, 1.6 μm, Phenomenex, Torrance, CA, USA). Flexar FX10 ultra-performance liquid chromatograph (PerkinElmer, Waltham, MA, USA) coupled with QTRAP 5500 mass spectrometer (Sciex, Redwood City, CA, USA) was used for the measurements. To perform quantitative analysis, multiple reaction monitoring was applied. For LC–MS analyses, we performed two independent experimental measurements with a minimum of three parallel cultures, and additionally minimum three measured samplings.

### 4.7. Statistical Analysis

Statistical analysis was performed using IBM SPSS Statistics software (version 22; SPSS Inc., Chicago, IL, USA), R (version 3.5.3), and RStudio (version 1.2.1335; RStudio Inc., Boston, MA, USA). For parametric data, two-sample t-test and one-way ANOVA with Tukey’s post hoc test were applied. Non-parametric data were analyzed with Mann–Whitney U test. Chi-square test and Fisher’s exact test were used to compare categorical variables. Correlations were assessed using Spearman’s rank correlation. Two-sided *p* values were considered statistically significant below 0.05.

## Figures and Tables

**Table 1:** Clinicopathological Data.

	IHC Cohort		TCGA Cohort	
Parameters	Papillary Renal Cell Carcinoma N (%)	Clear Cell Renal Cell Carcinoma N (%)	Papillary Renal Cell Carcinoma N (%)	Clear Cell Renal Cell Carcinoma N (%)
Age (years)				
≤65	16 (80)	15 (60)	170 (62)	332 (66)
>65	4 (20)	10 (40)	104 (38)	170 (34)
Gender				
Male	13 (65)	17 (68)	208 (75)	322 (64)
Female	7 (35)	8 (32)	69 (25)	180 (36)
pT Stage				
pT1	14 (70)	13 (52)	187 (68)	248 (50)
pT2	3 (15)	6 (24)	32 (12)	64 (13)
pT3	2 (10)	6 (24)	53 (19)	177 (35)
pT4	1 (5)	0 (0)	2 (<1)	11 (2)
Nuclear Grade				
1–2	16 (80)	17 (68)	N.A.	223 (45)
3–4	4 (20)	8 (32)	N.A.	271 (55)
Transplantation				
Post-transplant *	4 (20)	2 (8)	N.A.	N.A.
Non-transplant	16 (80)	23 (92)	N.A.	N.A.

* Four patients received tacrolimus and two patients received cyclosporine as immunosuppressive therapy. Abbreviations: IHC, immunohistochemistry; N.A., not available; pT, pathological T stage; TCGA, The Cancer Genome Atlas.

**Table 2:** Antibodies used for Protein Expression Analyses.

Protein Size (kDa)	IHC Dilution	Clone	Catalog No.	Supplier	Significance	Antibody
289	–	–	#2971	Cell Signaling	Active mTOR kinase	Anti-phospho(Ser2448)-mTOR
289	0.1111111	49F9	#2976	Cell Signaling	Active mTOR kinase	Anti-phospho(Ser2448)-mTOR
289	–	7C10	#2983	Cell Signaling	Amount of mTOR protein	Anti-mTOR
32	0.1111111	–	#2211	Cell Signaling	mTORC1 activity	Anti-phospho(Ser235/236)-S6
60	–	D9E	#4060	Cell Signaling	mTORC2 activity	Anti-phospho(Ser473)-Akt
60	–	C67E7	#4691	Cell Signaling	Amount of Akt protein	Anti-Akt (pan)
200	–	–	#2140	Cell Signaling	Amount of mTORC2	Anti-Rictor
200	0.7361111	1G3P2C9	A500-002	Bethyl	Amount of mTORC2	Anti-Rictor
55	0.3194444	–	ab652	Abcam	Glucose uptake	Anti-GLUT1
102	–	C35C4	#2024	Cell Signaling	Glycolysis	Anti-HXK1
102	0.1805556	C64G5	#2867	Cell Signaling	Glycolysis	Anti-HXK2
80	0.1111111	D4B2	#8164	Cell Signaling	Glycolysis	Anti-PFKP
37	–	C4B5	#3582	Cell Signaling	Warburg effect	Anti-LDHA
37	–	60H11	ab85319	Abcam	Reverse Warburg effect	Anti-LDHB
59	0.1111111	EPR6292	ab133525	Abcam	Pentose phosphate pathway	Anti-G6PD
55–60	0.1805556	EP7212	ab156876	Abcam	Glutaminolysis	Anti-GLS
78	0.1805556	D19C6	#3658	Cell Signaling	Acetate utilization	Anti-ACSS2
88	0.3888889	8F6AE9	ab128568	Abcam	β-oxidation of fatty acids	Anti-CPT1A
52	0.1111111	3D5	ab14730	Abcam	Oxidative phosphorylation	Anti-ATPB
17	1.4305556	3E11	#4850	Cell signaling	Terminal oxidation	Anti-COX IV
16	0.1111111	D8T4N	#42406	Cell Signaling	Mitochondrial marker	Anti-TOM20
45	–	AC-74	A2228	Sigma-Aldrich	Loading control	Anti-β-actin

Abbreviations: ACSS2, acetyl-coenzyme A synthetase short-chain family member 2; Akt, protein kinase B; ATPB, β-F1-ATPase; COX IV, cytochrome c oxidase complex IV; CPT1A, carnitine palmitoyltransferase 1A; G6PD, glucose-6-phosphate dehydrogenase; GLS, glutaminase; GLUT1, glucose transporter 1; HXK1, hexokinase 1; HXK2, hexokinase 2; IHC, immunohistochemistry; LDHA, lactate dehydrogenase A; LDHB, lactate dehydrogenase B; mTOR, mammalian target of rapamycin; PFKP, phosphofructokinase; Rictor, rapamycin-insensitive companion of mTOR; TOM20, translocase of the outer mitochondrial membrane complex subunit 20; WES, Wes^TM^ Simple analysis.

## Data Availability

The mRNA data presented in this study are deposited in TCGA repository (TCGA KIRC and TCGA KIRP datasets) and were accessed by cBioPortal (http://www.cbioportal.org, accessed on 15 July 2019).

## Supplementary Materials

The following supporting information can be downloaded at: https://www.mdpi.com/article/10.3390/ijms231810587/s1.
