# Supplementary figures and images for: Metabolic Adaptation as Potential Target in Papillary Renal Cell Carcinomas Based on Their In Situ Metabolic Characteristics
Source: Int J Mol Sci. 2022 Sep 13;23(18):10587. doi: 10.3390/ijms231810587 (PMC9503093; doi:10.3390/ijms231810587)

Figure S1. Additional information to WES Analysis

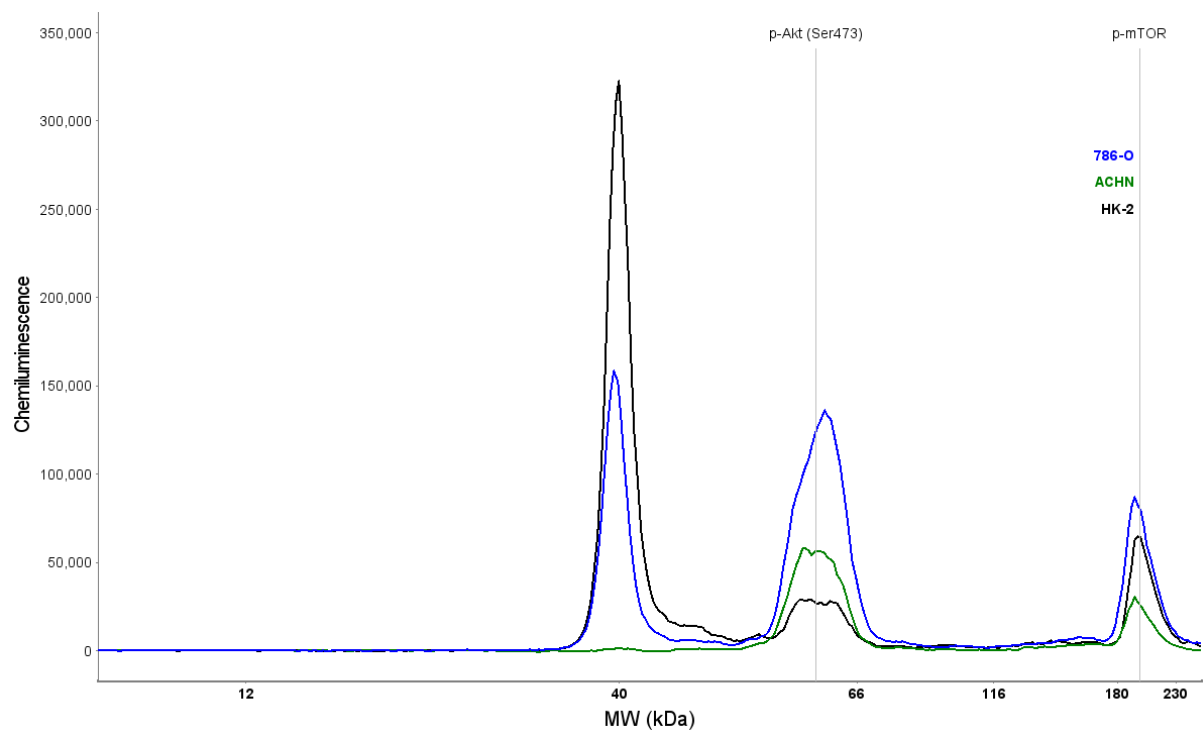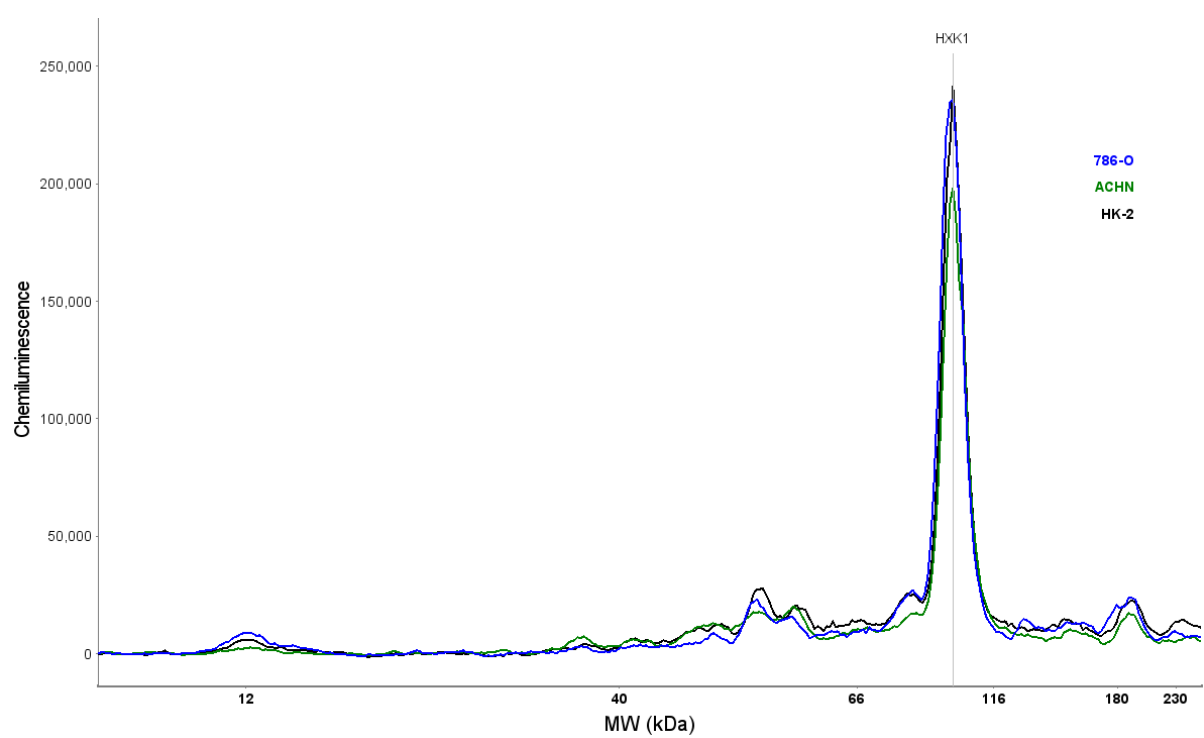

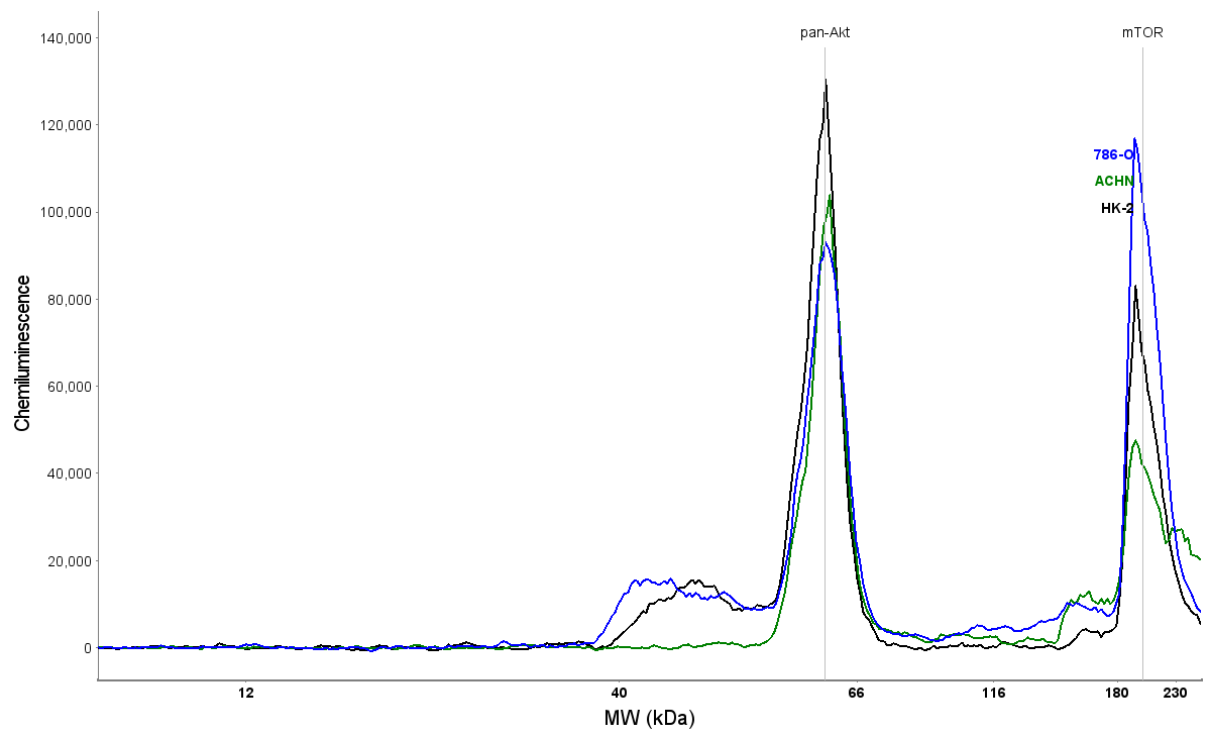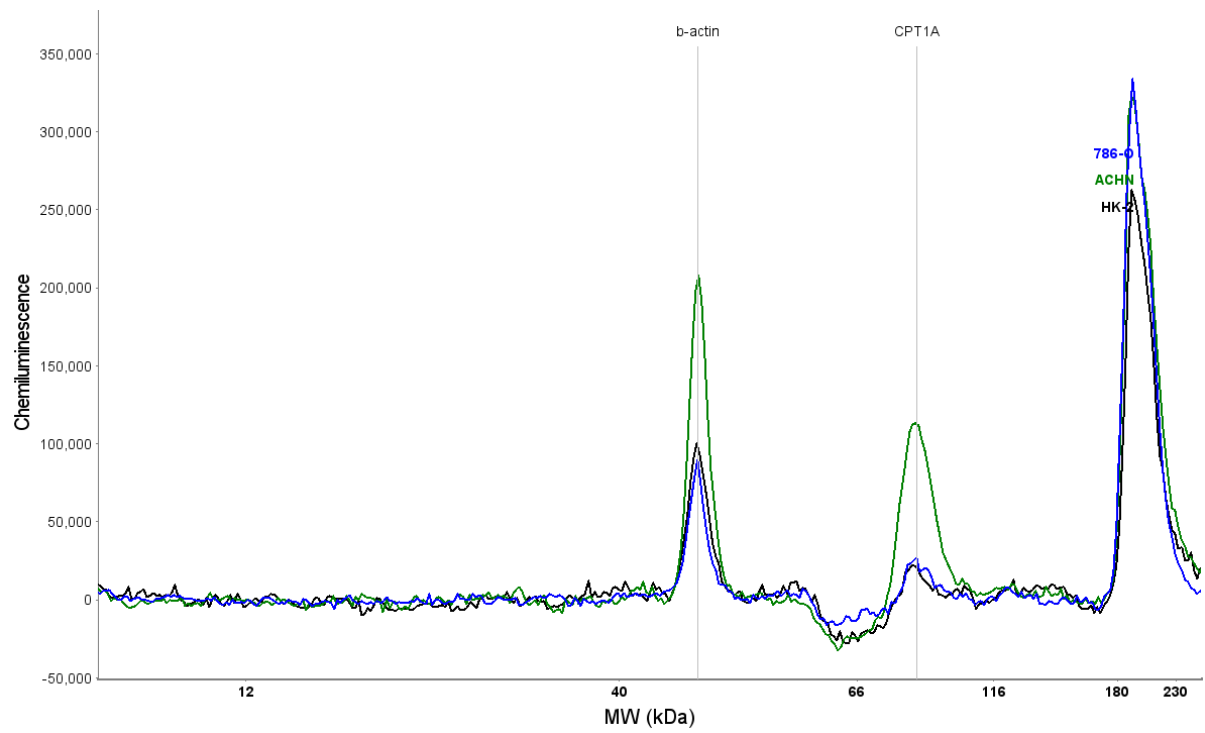

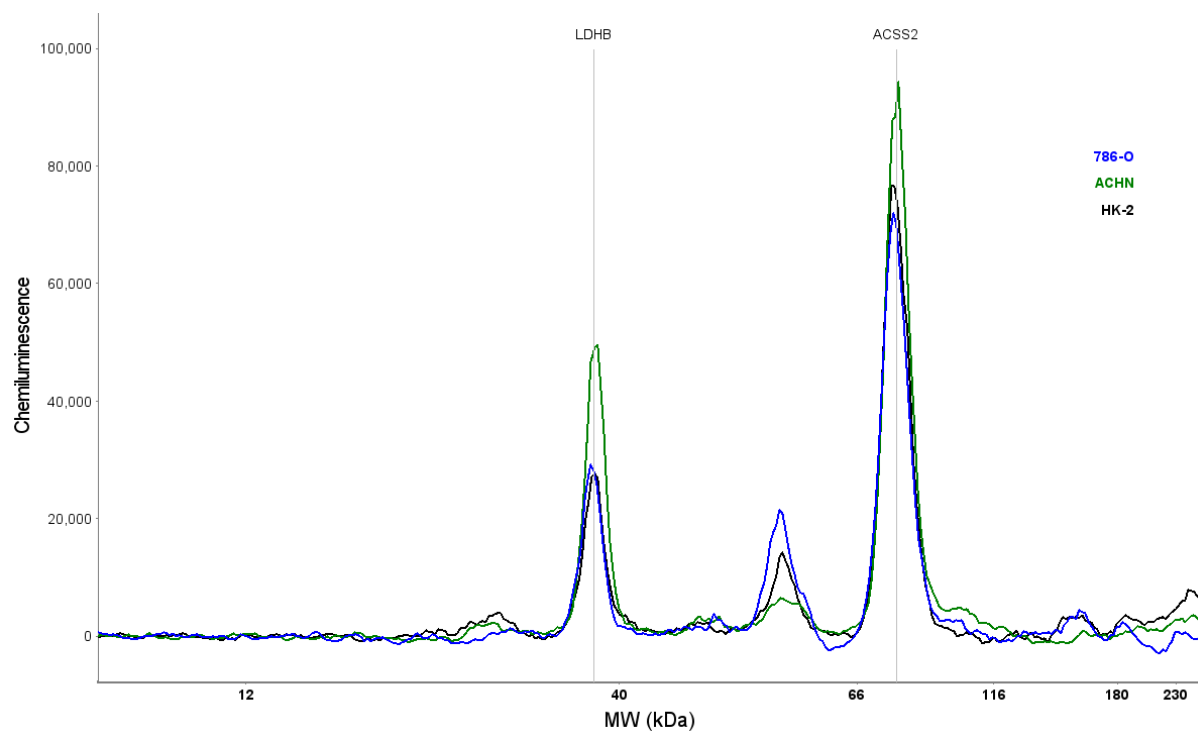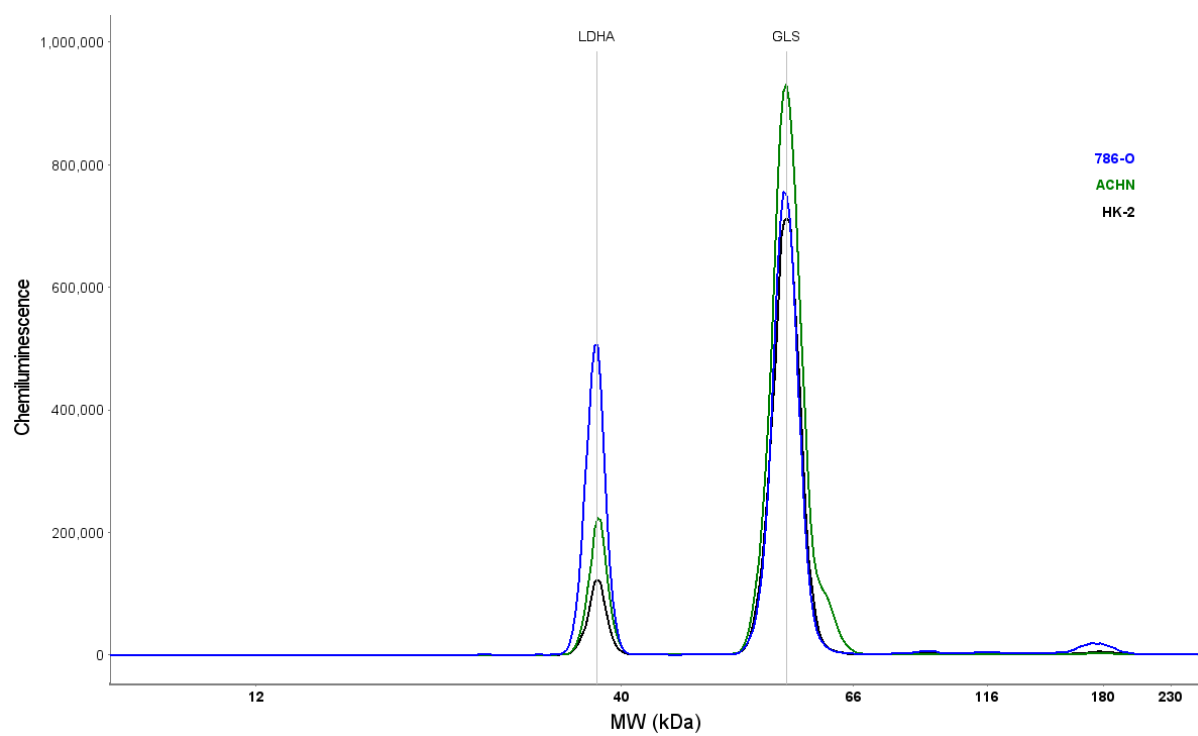

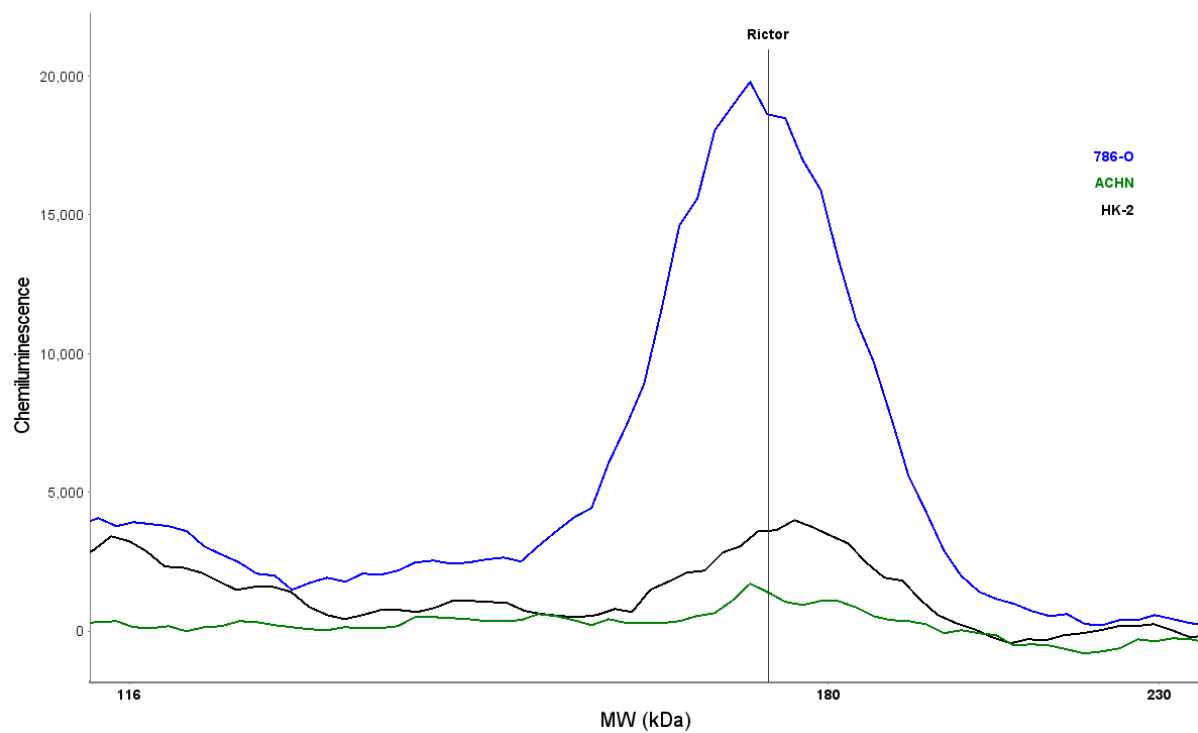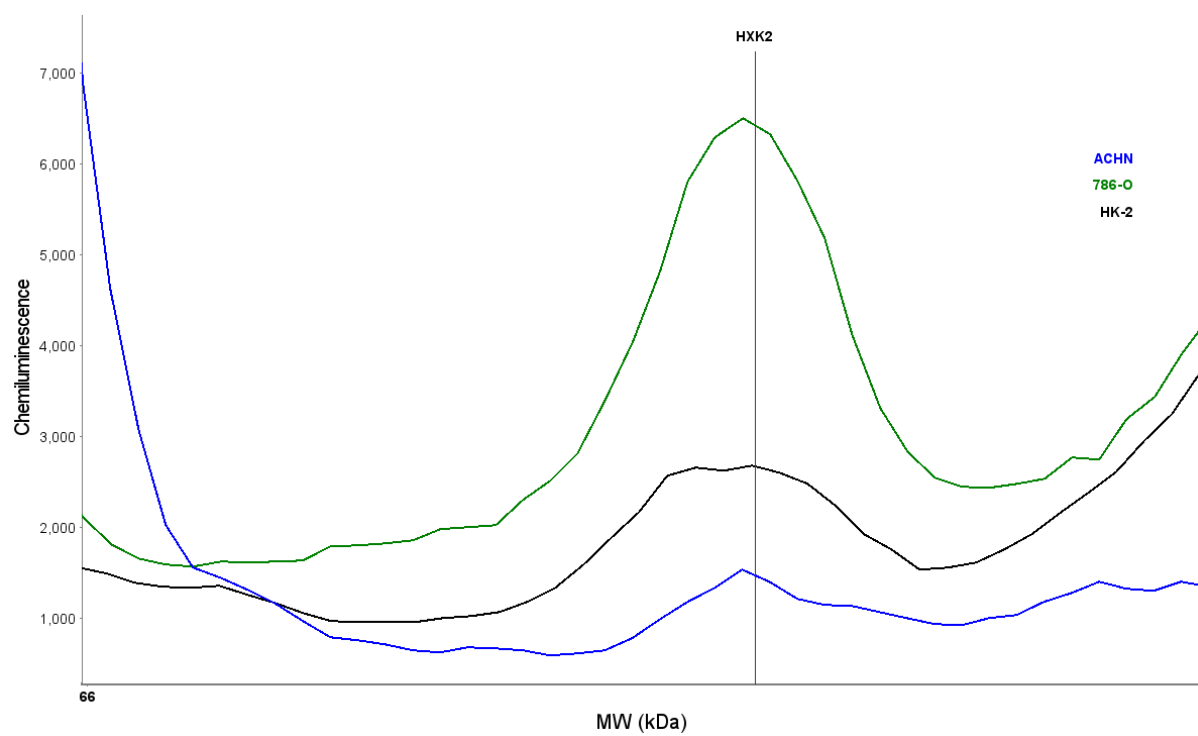

Supplement: Supplementary file 1 [file ijms-23-10587-s001.zip › ijms-1814851-supplementary.pdf]
